# Sterility and oxygenator function in pre-primed extracorporeal membrane oxygenation: A prospective clinical study

**DOI:** 10.1016/j.resplu.2024.100680

**Published:** 2024-06-05

**Authors:** Daniel Bengtsson, Bodil Jönsson, Bengt Redfors

**Affiliations:** a Department of Perfusion, Sahlgrenska University Hospital, Gothenburg, Sweden; b Institute of Biomedicine, Department of Infectious Diseases, Sahlgrenska Academy, University of Gothenburg, Gothenburg, Sweden; c Region Västra Götaland, Sahlgrenska University Hospital, Department of Clinical Microbiology, Gothenburg, Sweden; d Department of Cardiothoracic Anesthesia and Intensive Care, Sahlgrenska University Hospital, Gothenburg, Sweden; e Department of Anesthesiology and Intensive Care Medicine, Sahlgrenska Academy, University of Gothenburg, Gothenburg, Sweden

**Keywords:** Extracorporeal Membrane Oxygenation, Membrane Oxygenators, Equipment Contamination

## Abstract

**Aim:**

Minimizing cardiac arrest times is critical in extracorporeal cardiopulmonary resuscitation (ECPR). Pre-primed extra corporeal membrane oxygenator (ECMO) is used for this, but knowledge is limited to experimental studies. We prospectively investigated oxygenator function and sterility in dry plus wet pre-priming in a clinical setting.

**Methods:**

This prospective clinical study included 107 ECMO circuits used at Sahlgrenska University Hospital between October 2019 and December 2021. Circuits underwent dry set-up, followed by wet priming when the previous wet-primed circuit was used. Sterility was assessed by culturing the priming solution. Oxygenator function parameters, including sweep gas flow, fraction of oxygen (FiO_2_), and oxygenator resistance, were analyzed at ECMO initiation and during treatment using linear mixed models.

**Results:**

Median total set-up time was 14 days (range 0–97), with a median wet prime time of 6 days (range 0–57). 103 of 105 circuits with culture results were negative, two showed bacterial growth (coagulase-negative staphylococci and *Cutibacterium acnes*). Wet prime time did not significantly affect initial oxygenator function. Oxygenator resistance and FiO_2_ increased during ECMO treatment (0.035 mmHg/L min^−1^ (95 % confidence interval (CI) 0.015–0.055) *P* < 0.001, and 2.19 % (0.92–3.46) *P* = 0.009), but these changes were unrelated to wet prime time.

**Conclusion:**

Wet pre-priming of ECMO circuits for up to 57 days did not affect oxygenator function. The low incidence of bacterial growth (1.9 %) suggests that pre-primed ECMO generally maintain sterility and can facilitate rapid ECPR initiation. However, bacterial growth highlights the need for caution in non-urgent cases. Culturing the circuit at initiation can mitigate this risk.

## Highlights

• Pre-primed ECMO can decrease CPR times by enabling rapid ECPR initiation.
• Wet pre-prime duration of ECMO does not affect oxygenator function.
• Only 2 out of 105 pre-primed ECMO circuits showed bacterial growth.
• The low infection rate supports ECPR use but implies caution in non-urgent ECMO.
• Culturing ECMO circuits at initiation can guide treatment and enhance patient safety.

## Introduction

Survival rates in refractory cardiac arrest are poor, with less than 1 % of patients surviving with good neurological outcomes after 20 min of cardiopulmonary resuscitation.^1^ Extracorporeal cardiopulmonary resuscitation (ECPR), which utilizes an extracorporeal membrane oxygenation (ECMO) device to restore circulation, is increasingly used and included in international guidelines to improve survival in selected patients with refractory cardiac arrest.^2^, ^3^, ^4^ In ECPR, minimizing the cardiac arrest time — the time before a primed ECMO device restores circulation — is crucial for improving survival rates.^2^, ^4^, ^5^ However, the set-up and priming process of ECMO is time-consuming and can significantly prolong the cardiac arrest time, thereby delaying ECMO initiation.

To minimize the time to ECMO initiation during cardiac arrest, pre-priming the ECMO device in advance is an option.^6^ However, this practice is not recommended by manufacturers. For instance, the Cardiohelp (Getinge AB, Gothenburg, Sweden) manual states, “*only fill the system shortly before use*” and warns that “*a system that is filled too early (pre-priming) can lead to bacterial growth in the system and infections in the patient*”.^7^ This recommendation is consistent with guidelines for other intravenous fluids, which stipulate that fluids should be hung for only a short time after spiking/priming to minimize the risk of bacterial growth.^8^ However, as pre-priming may be advantageous for patient outcomes in ECPR, the Extracorporeal Life Support Organization (ELSO) suggests that ECMO can be pre-primed, but not for longer than 30 days, despite the manufacturer recommendations.^9^

The scientific basis for pre-primed ECMO is based on a limited number of experimental studies, each involving a small number of ECMO circuits with wet pre-primed times ranging from 7 to 65 days.^10^, ^11^, ^12^, ^13^, ^14^, ^15^ One study incorporated a dry set up phase prior to wet priming.^10^ Although no bacterial growth was observed in any of the circuits, it is important to note that the reported incubation times, during which bacteria and fungi can proliferate, were only two or three days when mentioned, while the general standard for considering cultures negative requires an incubation period of at least 5 days.^16^ In two of the studies, a few ECMO circles were wet-primed and inoculated with *Escherichia coli*, resulting in the persistence of bacteria within the circuits for an extended period.^12^, ^14^

Wet pre-priming could potentially damage the polymethylpentene (PMP) fibers of the oxygenator or create a vulnerability, which may manifest as decreased oxygenator gas exchange efficiency or increased flow resistance.^17^ These effects could be observed immediately at ECMO initiation or evolve during treatment. Three of the experimental studies assessed oxygenator function after wet priming and observed decreased oxygenation or carbon dioxide (CO_2_) removal from the test solution during short test periods.^10^, ^11^, ^15^

Thus, ECPR has the potential to improve survival in cardiac arrest patients, and pre-primed ECMO is valuable in decreasing cardiac arrest times. However, the scientific evidence supporting the sterility and oxygenator function of pre-primed ECMO is limited to controlled experimental setups, which may not accurately reflect the challenges encountered in clinical settings.

Consequently, this prospective clinical study was designed to investigate the effects of dry plus wet pre-priming and storage time on ECMO oxygenator function and sterility in a clinical environment.

## Methods

This prospective clinical trial included the ECMO circuits used at Sahlgrenska University Hospital between October 2019 and December 2021. The study was approved by the Regional Ethics Committee of Gothenburg (Dnr id 288-17, Dnr 2020-02043) and registered in Clinical Trials.gov (NCT04198792).

### ECMO and priming

All ECMO circuits were Cardiohelp System with HLS Set Advanced 7.0. Two Cardiohelp systems were always set up: one dry and one wet-primed. When the wet-primed circuit was used, the dry circuit was primed, and a new dry circuit was set up.

European Board Certified Clinical Perfusionists (ECCP) performed all set-ups and wet primings according to the manufacturer's instructions. The priming solution consisted of 1500–2000 ml Ringer-Acetat Baxter Viaflo (Baxter Medical, Kista, Sweden), with each 1000 ml containing 5.86 g sodium chloride, 0.30 g potassium chloride, 0.29 g calcium chloride dihydrate, 0.20 g magnesium chloride hexahydrate, and 4.08 g sodium acetate trihydrate (osmolality: 277 mOsm l^−1^, pH: 5.0–6.0). The ECMO circuit held 600 ml of the priming solution, and the remainder was stored in the priming bag. After priming, the ECMO circuit was turned off, stored with the power, sweep gas, and heater-cooler switched off, covered with plastics, and kept in the cardiothoracic operating suite.

At the time of use, the ECMO system was turned on, and the prime solution was circulated at 1500 rpm for 2–3 min. Before cannulation, patients received unfractionated heparin (UFH) 100 IU kg^−1^ or a fixed dose of 10,000 IE if the weight was unclear. Patients were cannulated using Maquet HLS Cannulae, 19–23 Fr (Getinge AB, Gothenburg, Sweden), based on size and clinical requirements. After ECMO initiation, the priming bag and tubing were disconnected and sent for sterility testing.

### Patients

All patients receiving ECMO treatment were managed according to strict local guidelines. The anticoagulation protocol included continuous intravenous heparin infusion, targeting an activated partial thromboplastin time (APTT) level of 50 to 70 s, but adjusted individually based on the patient's risk of bleeding and coagulation disorders.

### Data collection

The total times of dry and wet set-up were recorded for each ECMO circuit. To evaluate oxygenator performance, sweep gas flow (L min^−1^), fraction of oxygen in the sweep gas flow (FiO_2_ %), and oxygenator resistance (defined as the oxygenator blood pressure drop per liter of blood per minute, delta mmHg/L min^−1^) were analyzed. These oxygenator function parameters were measured at specific time points: immediately after ECMO initiation (0 h), 12 h after initiation, 24 h after initiation, and then daily until the end of the ECMO treatment. To assess the impact of wet-priming duration on oxygenator performance, the oxygenators were grouped based on the duration of wet-priming before use, forming four wet prime time groups: 0–1 day, 2–4 days, 5–9 days, and ≥ 10 days.

### Sterility control

A sample of 30–40 mL of prime solution was inserted into a 50 mL sterile test tube (Falcon, World Wide Range (WWR), Spånga, Sweden). This solution was used for the sterility control. The solution was inoculated into each of four culturing flasks with different media: 85 mL Thioglycollate Agar (TA(S)), 45 mL tryptic soy broth (TSB) at 37 °C, 45 mL TSB at 20 °C, and 45 mL malt extract broth (MEB). 5 mL of prime solution was added to each flask. The TA(S) and TSB 37° media were incubated at 37 °C for 7 days, while the TSB 20° and MEB media were incubated at 20 °C for 7 and 21 days, respectively. The flasks were checked for growth (haze) every working day. Negative flasks were reported as “Negative” after 7 days, but incubation continued until day 21, with late growths denoted in additional reports. The final result of growth is used in the study.

Microscopy of Gram-stained samples was used to determine the presence of Gram-positive or Gram-negative bacteria. Positive TAS flasks were inoculated on horse blood agar (aerobic, 2 days) and fastidious anaerobe agar (FAA) (anaerobic, 2 days) plates. TSB flasks were inoculated on horse blood agar plates (2 days), and MEB flasks on horse blood agar (2 days) and Sabouraud agar (yeast and molds, 3 days) plates. Bacteria were identified to species level with MALDI-TOF. Yeast and molds were identified as such, with species identification if requested. All isolated microorganisms were stored at −70 °C. Bags with remaining prime solution were stored at + 4 °C until sterile control was finished.

## Statistics

Continuous variables are presented as mean ± SD, with *P* < 0.05 considered statistically significant. Due to local protocol endorsing 100 % FiO_2_ for patients on venovenous ECMO (VV-ECMO), only patients on venoarterial ECMO (VA-ECMO) were included in the statistical analysis of FiO_2_.

Linear mixed models (LMM) were used to analyze the data, with ECMO run time as a repeated measure for each subject. The models included individual subjects as a random effect and used a compound symmetry covariance structure. LMMs assessed the impact of wet prime time group on the initial values of sweep gas flow, FiO_2_, and oxygenator resistance, as well as the effect of ECMO run time on these parameters. A multivariable LMM evaluated the combined effect of wet prime time group, ECMO run time, and their interaction on the three oxygenator performance parameters.

Model residuals were assessed for normality. Sweep gas flow and FiO_2_ demonstrated normality, while oxygenator resistance showed a positive skew. A logarithmic transformation normalized oxygenator resistance model residuals, and the transformed variable was used in subsequent analyses.

All statistical analyses were conducted using SPSS Statistics 28 (IBM Corp., Armonk, NY, USA).

## Results

During the study period, 113 Cardiohelp circuits were utilized; however, six cases were excluded due to missed inclusions. Thus, 107 circuits were included in the study. Median total set-up time was 14 days (range 0–97), dry set-up time 6.5 days (range 0–89), and wet-prime time 6 days (range 0–57). Mean ECMO run was 3.1 days, median 1 (range 0–84). 100 treatments were VA-ECMO (49 ECPR and 51 other VA), and 7 were VV-ECMO. In the wet prime time groups, there were 19 circuits in the 0–1 day group, 24 circuits in the 2–4 day group, 32 circuits in the 5–9 day group, and 32 circuits in the ≥ 10 day group.

### Sterility

**Fig. 1:**
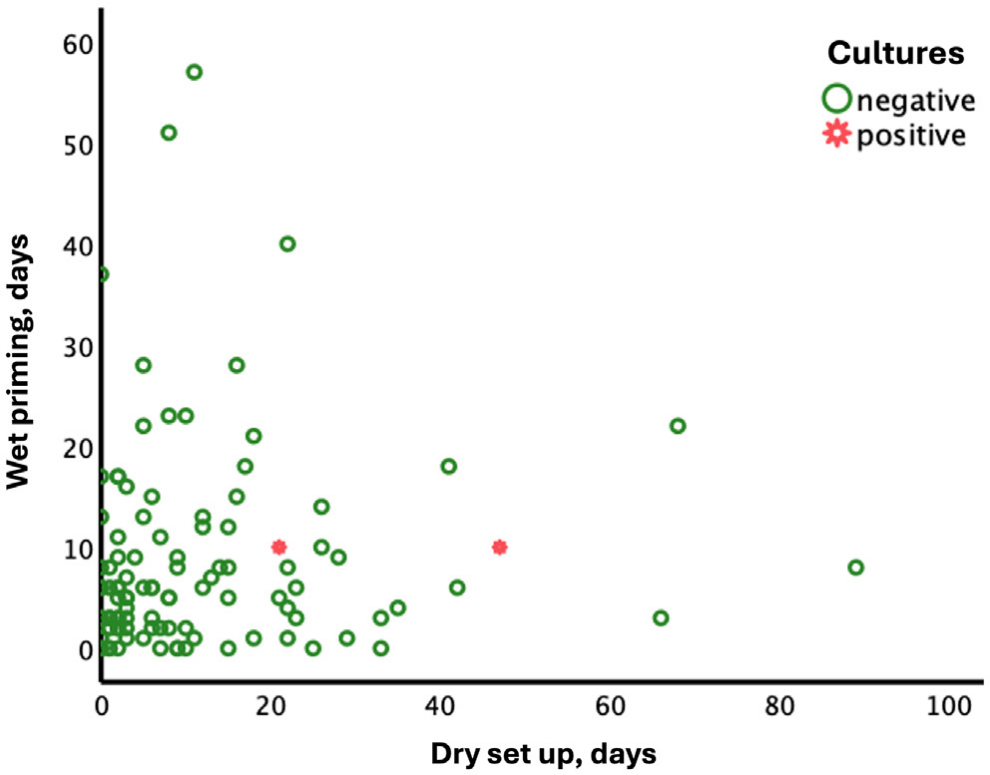
The result of the cultures. 103 circuits had negative cultures, while two showed growth. One circuit that had a dry set up of time 21 days and a wet prime time of ten days had coagulase-negative staphylococci. The other had a dry set up time of 47 days and a wet prime time of ten days and showed growth of Cutibacterium acnes.

There were missing values for two circuits. Thus, 105 were analyzed, of which 103 had negative cultures and two showed growth. One circuit, with a dry set up time of 21 days and a wet priming time of ten days, showed coagulase-negative staphylococci after three days (Fig. 1). An additional sample from the priming bag was analyzed and gave the same result. The patient was treated with piperacillin-tazobactam, with the addition of vancomycin and micafungin from day five after ECMO initiation. The two blood cultures taken were negative. The patient died on day six after ECMO initiation due to cerebral anoxia after ECPR. The other circuit, with a dry set up time of 47 days and a wet prime time of ten days, yielded growth of *Cutibacterium acnes* on day seven after ECMO initiation. The patient died on day three after ECMO initiation due to anoxic brain injury after ECPR. No blood culture was taken.

### Oxygenator resistance

**Fig. 2:**
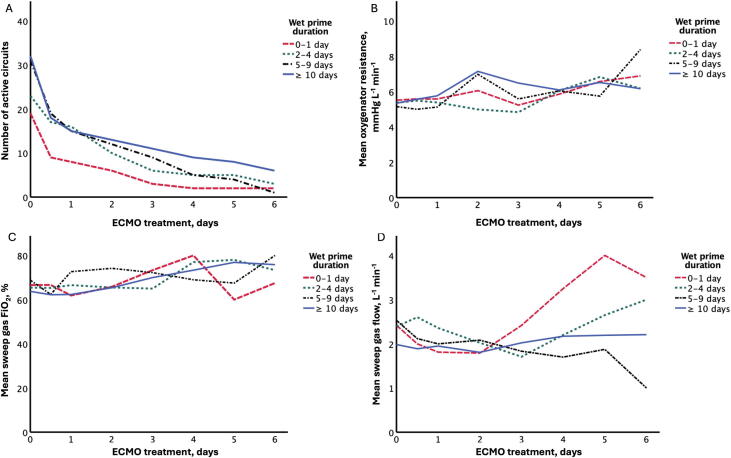
(A) Number of patients receiving ongoing ECMO treatment at various time points after ECMO initiation for the different wet prime time groups. Initially, there were 19 circuits in the 0–1 day group, 24 circuits in the 2–4 day group, 32 circuits in the 5–9 day group, and 32 circuits in the ≥ 10 day group. (B) Mean oxygenator resistance at each ECMO treatment time point for groups with different wet priming durations. No significant differences in oxygenator resistance were observed between the groups with varying wet priming times. (C) Mean fraction of inspired oxygen (FiO_2_) in the sweep gas flow for each group at different times of ECMO treatment. (D) Mean sweep gas flow for each group at different times of ECMO treatment. Similar to oxygenator resistance, no significant differences were found in FiO_2_ or sweep gas flow between the groups with different wet prime times. Furthermore, the changes in these parameters during ECMO treatment did not differ significantly between the groups.

The mean oxygenator resistance was 5,32 (±1.27) mmHg/L min^−1^ at ECMO-start, increasing to 6.47 (±1.44) mmHg/L min^−1^ after six days. This increase in resistance during ECMO run was significant (*P* < 0.001) (Table 1). However, there was no difference between the wet prime time groups in initial oxygenator resistance in univariate analysis. In multivariable analysis, ECMO run time still significantly affected the oxygenator resistance (*P* < 0.001), but resistance was not affected by wet prime time group. Additionally, the wet prime time group did not affect the increase in oxygenator resistance observed during the ECMO run, as shown by the lack of a significant effect of the interaction parameter (Fig. 2). Four ECMO circuits were changed in two patients due to high membrane pressure (n = 3) and hemolysis (n = 1). Wet prime times did not significantly differ between changed (11.7 ± 8.7 days) and unchanged circuits (8.6 ± 10.2 days).

**Table 1:** Oxygenator function parameters during the ECMO treatment.

	0 h	12 h	24 h	2 days	3 days	4 days	5 days	6 days	*P*
Oxygenator resistance, mmHg/L min^−1^ (n)	5.32 ± 1.27 (102)	5.37 ± 1.13 (63)	5.44 ± 1.23 (53)	6.41 ± 3.62 (41)	5.73 ± 2.18 (29)	6.04 ± 1.28 (21)	6.44 ± 1.37 (19)	6.47 ± 1.44 (12)	<0.001
FiO_2_, % (n)	64.1 ± 12.1 (97)	61.3 ± 13.1 (59)	63.9 ± 14.5 (50)	64.9 ± 15.9 (37)	65.6 ± 13.4 (25)	70.0 ± 13.8 (18)	70.9 ± 13.2 (16)	72.5 ± 7.2 (10)	<0.001
Sweep gas flow, L min^−1^ (n)	2.31 ± 0.95 (105)	2.17 ± 1.00 (63)	2.07 ± 1.15 (54)	1.94 ± 1.02 (41)	1.94 ± 1.13 (29)	2.171 ± .31 (21)	2.44 ± 1.48 (19)	2.52 ± 1.40 (12)	n.s.

Data are mean ± SD (n = number of measurements). *P*-values were calculated using linear mixed models (LMM), with ECMO run time as a repeated measure for each subject. A *P-*value < 0.05 was considered statistically significant.

Abbreviations: FiO_2_: Fraction of inspired oxygen in the sweep gas flow.

### FiO_2_

The mean FiO_2_ of the 100 VA-ECMO patients was 64.1 ± 12.1 % at ECMO-start increasing to 72.5 ± 7.2 % after 6 days. This increase was significant (*P* < 0.001). However, the wet prime time group did not affect initial FiO_2_ in univariate analysis. In multivariable analysis, FiO_2_ was still significantly affected by ECMO run time (*P* = 0.009), but neither the wet prime time group nor the interaction parameter was significantly affected.

### Gas flow

The mean gas flow was 2.31 ± 0.95 L min^−1^ at ECMO-start and 2.51 ± 1.10 L min^−1^ after six days. The change in gas flow during ECMO run was not found significant. The wet prime time group did not affect the initial sweep gas flow in the univariate analysis. In the multivariable analysis, neither ECMO run time, sweep gas flow group, nor their interaction affected gas flow.

## Discussion

In this prospective study, the first clinical investigation of pre-primed ECMO to our knowledge, we examined 107 ECMO circuits to assess the impact of wet prime time on sterility and oxygenator function. Sensitive sterility testing revealed a low incidence of bacterial growth (1.9 %) after dry set up for up to 90 days and wet priming up to 57 days, suggesting that sterility generally can be maintained for a prolonged period in a clinical setting. Regarding oxygenator function, wet prime time did not significantly influence oxygenator performance at the start of ECMO treatment. Although oxygenator resistance and FiO_2_ increased significantly during the ECMO run, these changes were not related to wet prime time. Together, this indicates a preserved integrity of the PMP fibers of the membrane oxygenator.

Pre-priming the ECMO circuit offers several advantages that must be weighed against the potential risks. Firstly, pre-priming allows for a faster initiation of ECMO support, which is critical in ECPR.^2^, ^6^ Secondly, it simplifies the starting process, thereby increasing the availability of this life-saving therapy. Moreover, pre-priming the ECMO circuit in a controlled environment can allow for a more meticulous and unhurried priming process, potentially reducing the risk of contamination compared to the rushed priming that may occur during ECPR cannulation. However, the risk of infection in the pre-primed circuit remains a concern, and the sterility of the ECMO circuit during treatment is of utmost importance. This was demonstrated by Kim et al., who found that bacterial colonization of the oxygenator reduced both weaning frequency and survival to one-third in 112 ECMO patients.^18^

This study adhered to ELSO's acceptance of general pre-priming of ECMO circuits and used pre-primed circuits for all patients.^9^ This minimises mean pre-prime times but exposes the patients who could have had unhurried ECMO priming to the risks of pre-priming. An alternative approach is using the pre-primed ECMO only in ECPR patients, ensuring that only those who benefit from shorter ECMO initiation times are exposed to the risks of pre-priming. However, this approach will lead to longer pre-priming times, particularly if ECPR patients are uncommon at the centre. In this study, the median wet pre-prime time of 6 days led to a low risk of contamination.

Experimental studies have demonstrated that bacteria can propagate and persist in ECMO circuits for extended periods when intentionally inoculated.^10^, ^12^ However, the crystalloid priming medium used in clinical practice lacks cellular substrates, and the room temperature storage of ECMO circuits may result in lower bacterial growth rates compared to body fluids, potentially posing challenges for sterility testing. Our study employed a sensitive culturing method specifically designed for extracorporeal fluids, involving extended incubation times. In contrast, previous experimental studies that reported no bacterial growth used shorter culturing durations of only two to three days or did not specify the duration.^10^, ^11^, ^12^, ^13^, ^14^

To ensure the sterility of the ECMO circuit, strict handling protocols must be followed to prevent the introduction of contaminants, particularly during the wet priming process, where the greatest risk of contamination can arise if the ECMO set's priming spike or the injection port of the crystalloid solution container is not completely sterile. This risk may be higher after dry set-up, as the spike has been outside the original sterile packaging during storage.

In our study, 103 of the 105 cultured ECMO circuits maintained sterility throughout the entire process, as evidenced by negative cultures. Two circuits yielded positive cultures. The coagulase-negative staphylococci growth in two separate samples taken from the priming bag at different time points strongly suggests an ECMO circuit infection. In contrast, the growth of *Cutibacterium acnes* after seven days indicates a low bacterial load, making it uncertain whether the result represents a true circuit infection or contamination during the sampling process. Notably, the two circuits with positive cultures had relatively long dry set-up times before wet priming, but further studies are needed to draw conclusions. The fact that 103 of the circuits remained sterile is reassuring, while the growth in two circuits underscores the importance of meticulous sterility during the pre-priming process. Culturing the ECMO circuit at initiation provides a simple measure to guide antibiotic treatment and serves as a patient safety system.

In addition to maintaining sterility, preserved ECMO function is equally important after wet priming. If the membrane oxygenator is negatively affected by pre-priming, it can either manifest immediately at ECMO initiation or lead to faster deterioration during ECMO treatment. The Cardiohelp HLS set oxygenator features hydrophobic PMP fibers coated with Bioline, a heparin and albumin coating designed to reduce protein adsorption and clotting activity.^19^, ^20^, ^21^ Damage to these fibers during wet pre priming could result in increased protein absorption, membrane fouling, thrombus formation, plasma leakage, and impaired oxygenator function.

Rather than directly measuring oxygenation and carbon dioxide removal through blood sampling on the oxygenator, we evaluated FiO_2_ levels, clinically required for adequate oxygenation of the patients, and sweep gas flow, clinically needed for carbon dioxide removal, from the patients. Notably, we found no significant differences in FiO_2_, gas flow, or oxygenator resistance between the groups of different wet-priming lengths, at ECMO initiation. This suggests that the wet pre-priming duration does not significantly impact the initial performance of the oxygenator. Furthermore, during ECMO treatment, we observed an increase in flow resistance and FiO_2_ need, which can be attributed to protein fouling and thrombus formation in the oxygenator.^21^ However, the magnitude of this increase was not related to pre-priming duration. Thus, oxygenator function deterioration during ECMO treatment was not affected by the wet priming duration. These results indicate that the PMP fibers and Bioline coating remained intact and were not compromised during the pre-priming period, ensuring consistent oxygenator function throughout ECMO treatment.

### Limitations

One limitation of this study is the relatively short mean ECMO run duration of 3.1 days (range 0–84), with all but one lasting 12 days or less. Consequently, the findings' validity regarding wet priming effects on oxygenator function is limited to this time frame. While our study found no effect of wet priming duration on clinically chosen FiO_2_ and sweep gas flow, directly measuring oxygenation and CO_2_ removal through blood gas analysis at the oxygenator inlet and outlet ports would provide a more precise assessment of oxygenator performance.

## Conclusions

In this prospective clinical study, we found that wet pre-priming of ECMO circuits for up to 57 days did not adversely affect oxygenator function. The low incidence of bacterial growth (2 out of 105 circuits) suggests that pre-primed ECMO generally maintain sterility and can be valuable in facilitating rapid ECPR initiation. However, the occurrence of bacterial growth emphasises the need for caution when using pre-primed ECMO for non-urgent cases. Implementing a protocol to culture the circuit at initiation can help mitigate these risks.

## Funding

The research was funded by the Swedish Heart-Lung Foundation [grant number 20200197] and the Swedish Society of Medicine [grant number SLS-935423].

## CRediT authorship contribution statement

**Daniel Bengtsson:** Writing – review & editing, Writing – original draft, Methodology, Investigation, Conceptualization. **Bodil Jönsson:** Writing – review & editing, Methodology, Investigation, Conceptualization. **Bengt Redfors:** Writing – review & editing, Writing – original draft, Methodology, Formal analysis, Data curation, Conceptualization.

## Declaration of competing interest

The authors declare the following financial interests/personal relationships which may be considered as potential competing interests: Bengt Redfors reports financial support was provided by The Swedish Heart-Lung Foundation. Bengt Redfors reports financial support was provided by The Swedish Society of Medicine. If there are other authors, they declare that they have no known competing financial interests or personal relationships that could have appeared to influence the work reported in this paper.
